# Protocol for rapid clearing and staining of fixed Arabidopsis ovules for improved imaging by confocal laser scanning microscopy

**DOI:** 10.1186/s13007-019-0505-x

**Published:** 2019-10-25

**Authors:** Rachele Tofanelli, Athul Vijayan, Sebastian Scholz, Kay Schneitz

**Affiliations:** 1 Entwicklungsbiologie der Pflanzen, Wissenschaftszentrum Weihenstephan, Technische Universität München, Emil-Ramann-Str. 4, 85354 Freising, Germany; 2 Present Address: EU Research Lab, Technische Hochschule Wildau, 15745 Wildau, Germany

**Keywords:** 3D reconstruction, 3D organ models, Arabidopsis, ClearSee, Imaging, Ovule, SCRI Renaissance 2200, To-PRO-3 iodide

## Abstract

**Background:**

A salient topic in developmental biology relates to the molecular and genetic mechanisms that underlie tissue morphogenesis. Modern quantitative approaches to this central question frequently involve digital cellular models of the organ or tissue under study. The ovules of the model species *Arabidopsis thaliana* have long been established as a model system for the study of organogenesis in plants. While ovule development in Arabidopsis can be followed by a variety of different imaging techniques, no experimental strategy presently exists that enables an easy and straightforward investigation of the morphology of internal tissues of the ovule with cellular resolution.

**Results:**

We developed a protocol for rapid and robust confocal microscopy of fixed Arabidopsis ovules of all stages. The method combines clearing of fixed ovules in ClearSee solution with marking the cell outline using the cell wall stain SCRI Renaissance 2200 and the nuclei with the stain TO-PRO-3 iodide. We further improved the microscopy by employing a homogenous immersion system aimed at minimizing refractive index differences. The method allows complete inspection of the cellular architecture even deep within the ovule. Using the new protocol we were able to generate digital three-dimensional models of ovules of various stages.

**Conclusions:**

The protocol enables the quick and reproducible imaging of fixed Arabidopsis ovules of all developmental stages. From the imaging data three-dimensional digital ovule models with cellular resolution can be rapidly generated using image analysis software, for example MorphographX. Such digital models will provide the foundation for a future quantitative analysis of ovule morphogenesis in a model species.

## Background

The ovule is the major female plant organ involved in sexual reproduction and the progenitor of the seed. In part due to their highly interesting biology the ovules of *Arabidopsis thaliana* also successfully serve as a general model system to study the molecular and genetic basis of plant organogenesis [1–5].

An individual Arabidopsis ovule primordium emerges as a finger-like protrusion emanating from the placental tissue of the gynoecium [6, 7]. Shortly thereafter, three distinct pattern elements can be recognized along the proximal–distal (PD) axis [6, 8]. At the distal end, the nucellus harbors a single subepidermal megaspore mother cell which will undergo meiosis and eventually form the haploid female gametophyte or embryo sac with the egg cell proper. The central chalaza is characterized by the two integuments that initiate at its flanks. The integuments are determinate lateral tissues that will eventually form the seed coat. Upon initiation they grow around the nucellus in an asymmetric fashion. The differential growth pattern of the integuments is a major determinant of the curved or anatropous shape of the mature ovule. Finally, the proximal funiculus is a stalk-like structure that connects the ovule to the placenta.

Several features of Arabidopsis ovules have facilitated genetic studies of their development, including their large number per gynoecium (around 50) and their relatively stereotypic development, allowing the thorough analysis of mutant phenotypes by easily scoring hundreds of ovules. Rapid and straightforward imaging of ovules represents a central aspect of such genetic studies. To this end several approaches are currently in use. Methods to investigate ovule morphology include scanning electron microscopy [7, 9–11], conventional light microscopy involving classical sectioning techniques or optical sectioning of hand-dissected ovules using organic clearing reagents, such as methyl benzoate [6] or Hoyer’s medium (which includes chloral hydrate as a clearing agent) [12, 13], confocal microscopy using fixed or live tissue [14–17], or optical projection tomography [18]. Moreover, gene expression can be studied using standard transgenic promoter–reporter-based approaches and by RNA in situ hybridization on sectioned [19] or whole-mount [20] tissue. Protein distribution and subcellular localization can also be assessed by reporter-based approaches or by immunostaining procedures [21, 22].

To gain a full understanding of the molecular and cellular basis of organogenesis it is essential to obtain a reliable three-dimensional (3D) representation of the architecture of an entire organ at different stages of development. Faithful digital 3D models with complete cellular resolution are essential prerequisites for quantitative analyses of the multiscale aspects of morphogenesis [23–25]. For example, they allow assessment of cell parameters, such as number, size, length, and number of neighbors. Such data enable quantitative morphometry which leads to an improved comparative analysis of wild-type and mutant phenotypes, and thus a deeper understanding of gene function. Cellular topology extracted from 3D digital models can be used to assess global emergent properties in tissues using network science [26]. Moreover, digital models can serve as templates to map patterns of gene expression patterns or subcellular protein localization in a tissue context. Furthermore, they are also essential as realistic tissue templates for modelling purposes.

Several approaches have been developed to image plant tissues [27, 28]. Ideally, live imaging is invoked in generating 3D representations of developing tissues. For example, deep live imaging using light sheet microscopy was employed to investigate the cell division patterns during early lateral root development [29, 30]. Live imaging has also been adopted when imaging tissue surfaces, such as the epidermis of the shoot apical meristem [31–33] or the sepal [34–37]. Due to technical reasons, however, the cellular analysis of deeper tissue layers usually involves fixed material. Pseudo-Schiff propidium iodide (PS-PI) has classically been used to stain cell walls in plant tissues [38]. A modified version of this technique (mPS-PI) involving fixed tissue followed by clearing using Hoyer’s medium has previously been established for high-resolution whole-mount imaging of 3D tissue organization with cellular resolution [16]. The mPS-PI method can be applied to ovules [11, 16, 39] and was used to generate 3D representations of the nucellus of several species, including *Arabidopsis thaliana* [39]. Moreover, the protocol allows for the analysis of gene expression patterns using promoter-GUS reporters [16, 40]. The method is unfortunately incompatible with imaging of fluorescent proteins (FPs) and thus does not allow the analysis of for example subcellular protein localization using FP-based fusion proteins.

New tissue clearing methods, including PEA-CLARITY [41], TOMEI [42, 43], and ClearSee [44], have recently been developed that allow deep imaging of plant tissues and are compatible with visualisation of various stains and FPs [45]. For example, ClearSee can be combined with several general cell wall stains [46], FPs, and imaging by confocal or two photon microscopy [44]. It can thus be used to analyze the 3D architecture of entire plant organs with cellular resolution and to investigate gene expression patterns and subcellular protein localization using respective reporters. In another important development the cell wall stain SCRI Renaissance 2200 (SR2200) was established as a convenient tool to mark the outline of cells in mature ovules, immature seeds and developing embryos and can also be combined with fluorescent reporters [17].

Here, we describe an improved method for imaging of ovules that is based on a combination of the ClearSee and SR2200 protocols. In addition, all nuclei, including the nuclei of the gametophyte, are stained with TO-PRO-3 iodide. It further comprises modifications that optimize the microscopy of Arabidopsis ovules. It thus allows rapid and reproducible deep imaging of the entire ovule at all developmental stages with cellular resolution. The resulting z-stacks allow rapid and robust segmentation followed by 3D digital model generation in software, such as MorphographX [47].

## Results

**Fig. 1:**
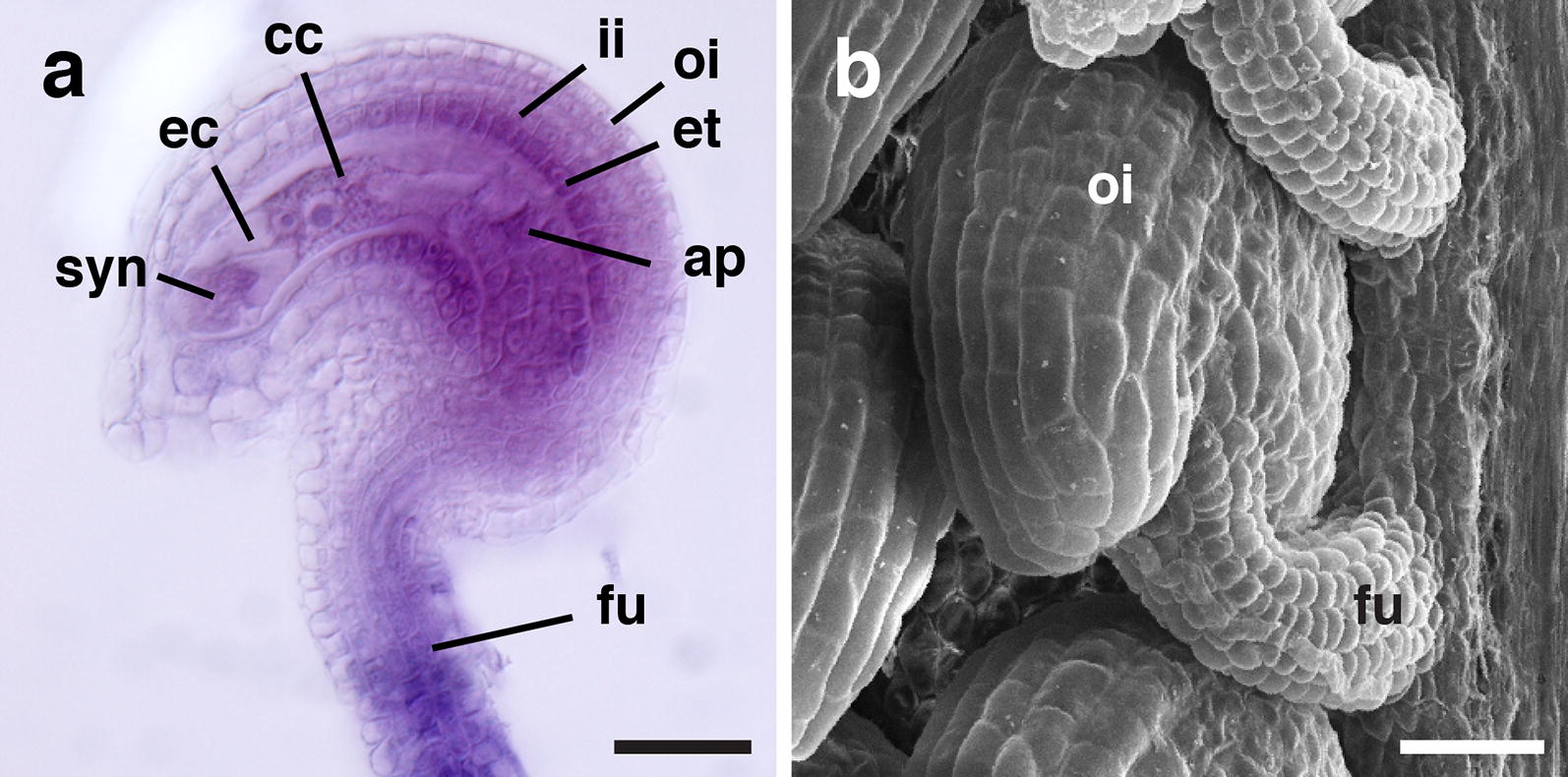
Micrographs of mature wild-type Arabidopsis ovules. **a** A mid-sagittal optical section of a stage 3-VI ovule. Clearing by methyl benzoate, Mayer’s hemalum staining, and imaging was done as described earlier [6, 73]. **b** Scanning electron micrograph of a stage 3-VI/4-I ovule. *ap* antipodal cells, *cc* central cell, *ec* egg cell, *et* endothelium, *fu* funiculus, *ii* inner integument, *oi* outer integument, *syn* synergid. Scale bars: 20 μm (Adapted from [73])

**Fig. 2:**
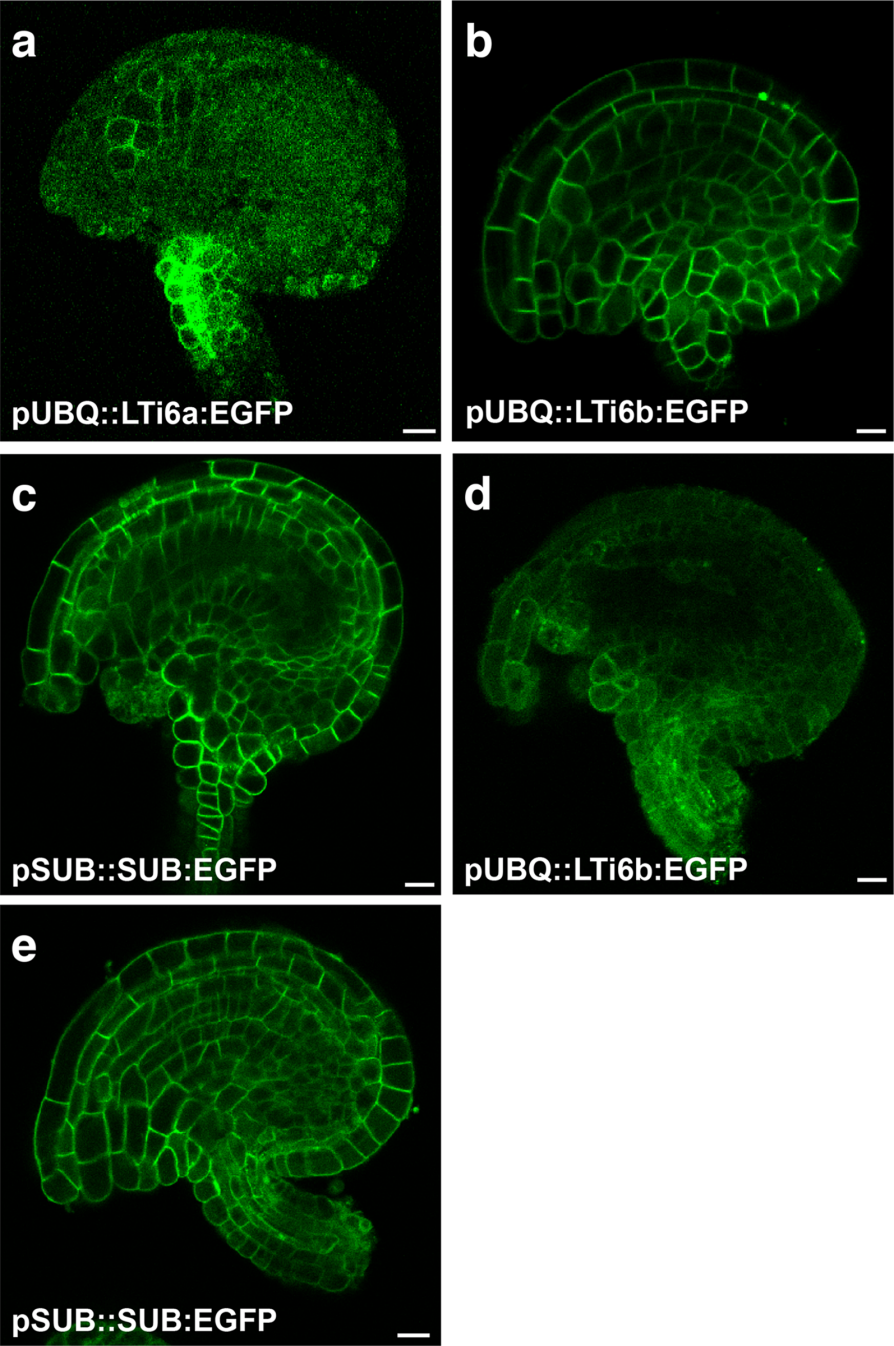
Confocal micrographs depicting mid-saggital optical sections of stage 3-V to 3-VI wild-type Arabidopsis ovules carrying fluorescent cell outline markers. **a** Live ovule expressing the pUBQ::LTi6a:GFP reporter. **b** Live ovule expressing the pUBQ::LTi6b:GFP reporter. **c** Live ovule expressing the pSUB::SUB:EGFP reporter. **d** Fixed ovule expressing the pUBQ::LTi6b:EGFP reporter. The ovule was fixed in 2% formaldehyde for 20 min. Compare to **b**. **e** Fixed ovule expressing the pSUB::SUB:EGFP reporter. The ovule was fixed in 2% formaldehyde for 20 min. Compare to **c**. Scale bars: 10 μm

We are interested in the molecular mechanisms underlying planar growth of integuments [48, 49] and thus we were particularly interested in live imaging of later-stage ovules that undergo integument outgrowth and curvature. Mature ovules with well-developed integuments can be visualized using classical techniques involving for example cleared and counterstained tissue or scanning electron microscopy (Fig. 1). However, those approaches mostly lack cellular resolution. To address this issue we initially focussed on confocal microscopy of live stage 3-V to 4-I ovules which are characterized by well-developed and curved integuments (Fig. 1) (stages according to [6]). To obtain cellular resolution we decided to mark the circumference of cells using a plasma membrane-localized FP. However, we encountered several problems. The first issue related to marking the cell outline of integument cells. To this end we tried to anchor an enhanced version of GFP (EGFP) [50] to the plasma membrane (PM). Interestingly, for as yet unknown reasons we observed that reporter lines carrying a transgene encoding a fusion of EGFP to LTI6a, a commonly used PM anchor [51, 52], under the control of the *UBIQUITIN10* promoter (*pUBQ::LTI6a:EGFP*) did not exhibit a signal at the PM of integument cells but diffuse signal was detected throughout the cytoplasm (Fig. 2a). Moreover, we noticed that integument cells of wave lines 131 (NPSN12:YFP) and 138 (PIP1;4:YFP) [53] did not show the expected PM-localized signal. By contrast, we found that reporter signal in integument cells of a line expressing GFP fused to LTI6b PM anchor [51] labelled the cell outlines, as expected (Fig. 2b). Similar results were obtained using the well-characterized pSUB::SUB:EGFP reporter [54] (Fig. 2c). *STRUBBELIG* (*SUB*) encodes a receptor kinase involved in tissue morphogenesis [54, 55]. The reason for the unexpected differential behavior between the various reporters is presently unknown but may perhaps relate to the extreme curvature of integument cells.

The dimensions of a typical mature Arabidopsis ovule (without the funiculus) are in the range of 105 µm (width) × 70 µm (height) × 55 µm (depth) (see below). Using standard confocal microscopy we could not perform live imaging through an entire mature ovule with cellular resolution due to light scattering and signal loss. We achieved a partial solution to this problem by applying a mild fixation step using 2% formaldehyde for 10 to 20 min. However, in fixed samples we noticed that the EGFP:LTI6b reporter signal showed prominent cytoplasmic localization (Fig. 2d). We did not observe a similar difference in signal distribution for the pSUB::SUB:EGFP reporter (Fig. 2e), however, loss of contrast and signal strength due to light scattering still pertained and proved prohibitive to imaging through the entire ovule with reliable cellular resolution.

Taken together, the results obtained so far indicated that the applied approaches were unsuitable for live imaging of later stages of ovule development with the necessary cellular resolution, in particular for deeper tissue layers. However, they also suggested that a strategy involving fixed ovules could prove more successful.

**Fig. 3:**
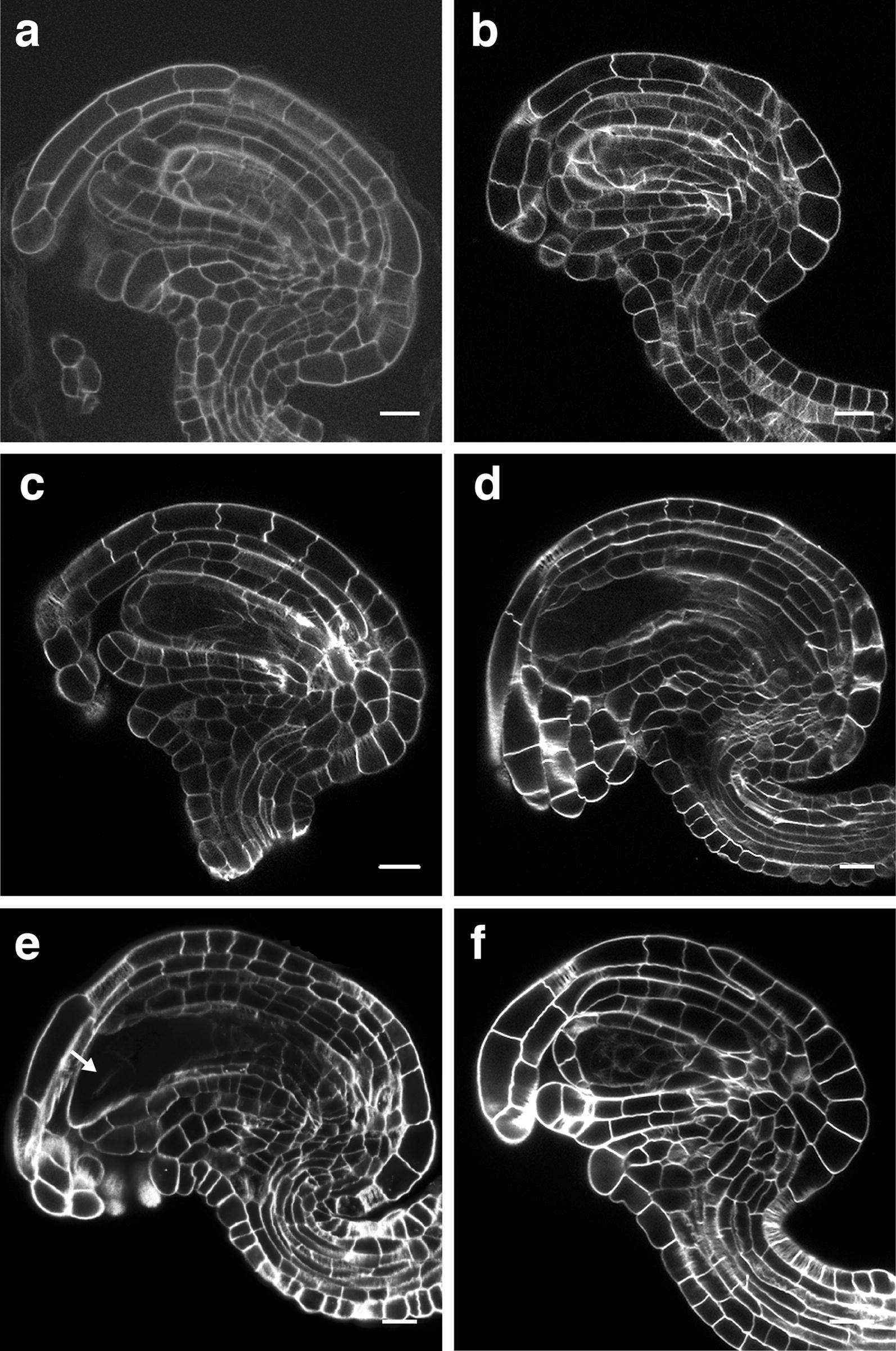
Confocal micrographs depicting mid-sagittal optical sections of fixed early stage 3 wild-type Arabidopsis ovules. **a** mPS-PI treatment. **b**–**d** Tissue cleared with ClearSee. **b** Direct Red 23 staining. **c** Calcofluor White staining. **d** SR2200 staining. **e** Fixed and SR2200-stained ovule with mature embryo sac. Note the weak or absent labelling of embryo sac cells. Arrow indicates weak signal at the synergids. **f** SR2200 staining in PBS followed by incubation in ClearSee solution. Scale bars: 10 μm

Therefore, we first applied the mPS-PI method to stain the cell walls. We could satisfactorily image through entire young primordia as well as mature pre-fertilization ovules with cellular resolution (Fig. 3a). However, while mPS-PI is an excellent tool for morphological studies, it is incompatible with fluorescent stains and FP-based reporters, therefore restricting its usefulness to approaches that do not require FPs. Thus, we decided to follow a different strategy and tested ovule clearing by the recently developed ClearSee method which can be combined with FPs and several cell wall stains [44, 46]. We dissected and fixed ovules of different developmental stages and cleared the tissue according to the ClearSee protocol. We then tested different cell wall-specific stains, including Direct Red 23, Calcofluor White, and SR2200 (Fig. 3b–d) [17, 46, 56–58]. We found that SR2200 is compatible with the ClearSee protocol. Importantly, we obtained the strongest, most robust, and reliable staining of cell outlines using SR2200. However, cell outlines of the cells of the mature embryo sac could not be stained reproducibly (Fig. 3e). This may in part be due to only partially formed cell walls in this tissue [59].

In previous work, cell wall staining was directly conducted in ClearSee [46]. In our hands, however, we observed somewhat higher background and consequently a reduction of the signal-to-noise (S/N) ratio when using SR2200 (Fig. 3d). By contrast, performing the SR2200 staining in PBS noticeably improved the S/N ratio and thus the overall quality of the obtained images (Fig. 3f). Thus, we now routinely transfer the cleared tissue to PBS, perform the staining with SR2200 followed by re-incubation with ClearSee for 20 min before mounting (see below).

**Fig. 4:**
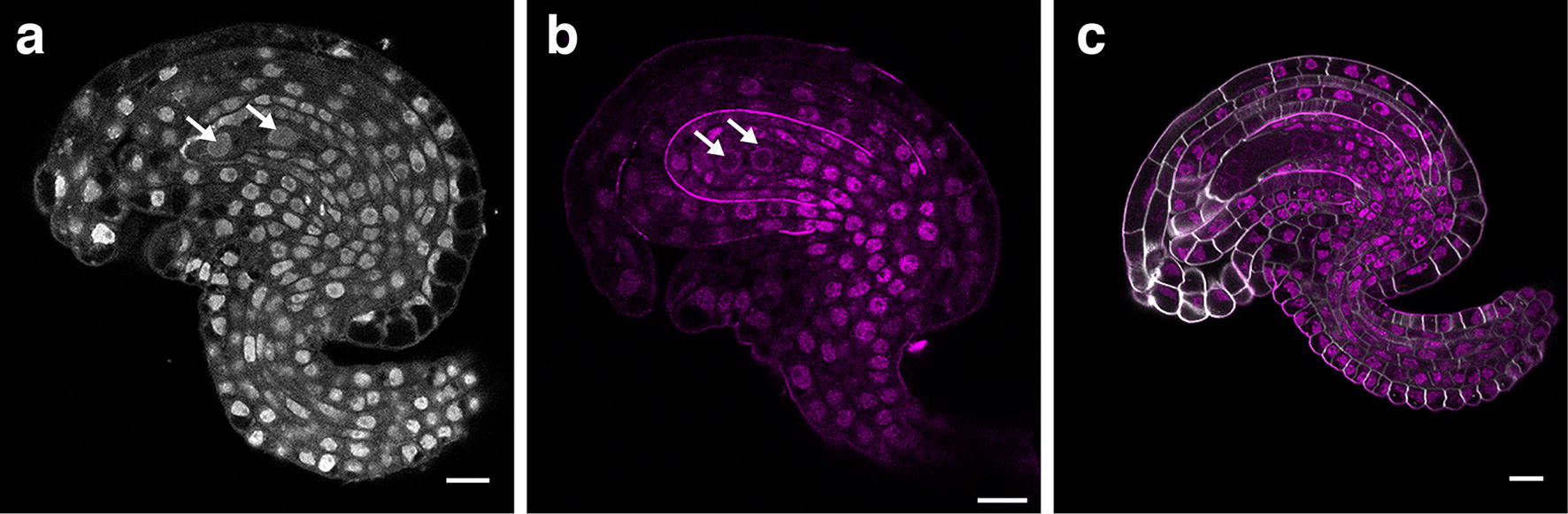
Nuclear staining of early stage 3 wild-type Arabidopsis ovules with TO-PRO-3 iodide. Confocal micrographs depict mid-sagittal optical sections. **a** DAPI treatment of an ovule exhibiting a two-nuclear embryo sac (stage 3-II [6]; stage FG2 [74]). Arrows indicate gametophyte nuclei. **b** TO-PRO-3 iodide treatment of an ovule exhibiting a two-nuclear embryo sac (stage 3-II [6]; stage FG2 [74]). Arrows indicate gametophyte nuclei. **c** An ovule carrying a four-nuclear embryo sac co-stained with SR2200 and TO-PRO-3 iodide (stage 3-IV [6]; stage FG4 [74]). Note the high-contrast signals labelling cell outlines and nuclei. Scale bars: 10 μm

Staging of Arabidopsis later-stage ovule development critically relies on the nuclear division pattern that is observed during the development of the haploid gametophyte [6, 14]. For unknown reasons, embryo sac nuclei are stained only weakly by conventional fluorescent nuclear stains, such as the Hoechst dye [60]. We noticed that the fluorescent nuclear stain TO-PRO-3 iodide (TO-PRO-3) [61, 62] conveniently stains nuclei in fixed ovules of all tested stages, including the nuclei of the developing embryo sac (Fig. 4b). However, nuclei of the embryo sac showed a weaker signal in comparison to the nuclei of the diploid cells surrounding the female gametophyte. Nevertheless, embryo sac nuclei can be observed and with an excitation maximum at 642 nm and an emission maximum at 661 nm the stain is compatible with SR2200-mediated cell wall labelling. In addition, TO-PRO-3 staining is performed in PBS, allowing us to combine the cell wall and nuclear staining using SR2200 and TO-PRO-3, respectively. This resulted in a less time-consuming staining procedure and reduced handling of samples.

**Fig. 5:**
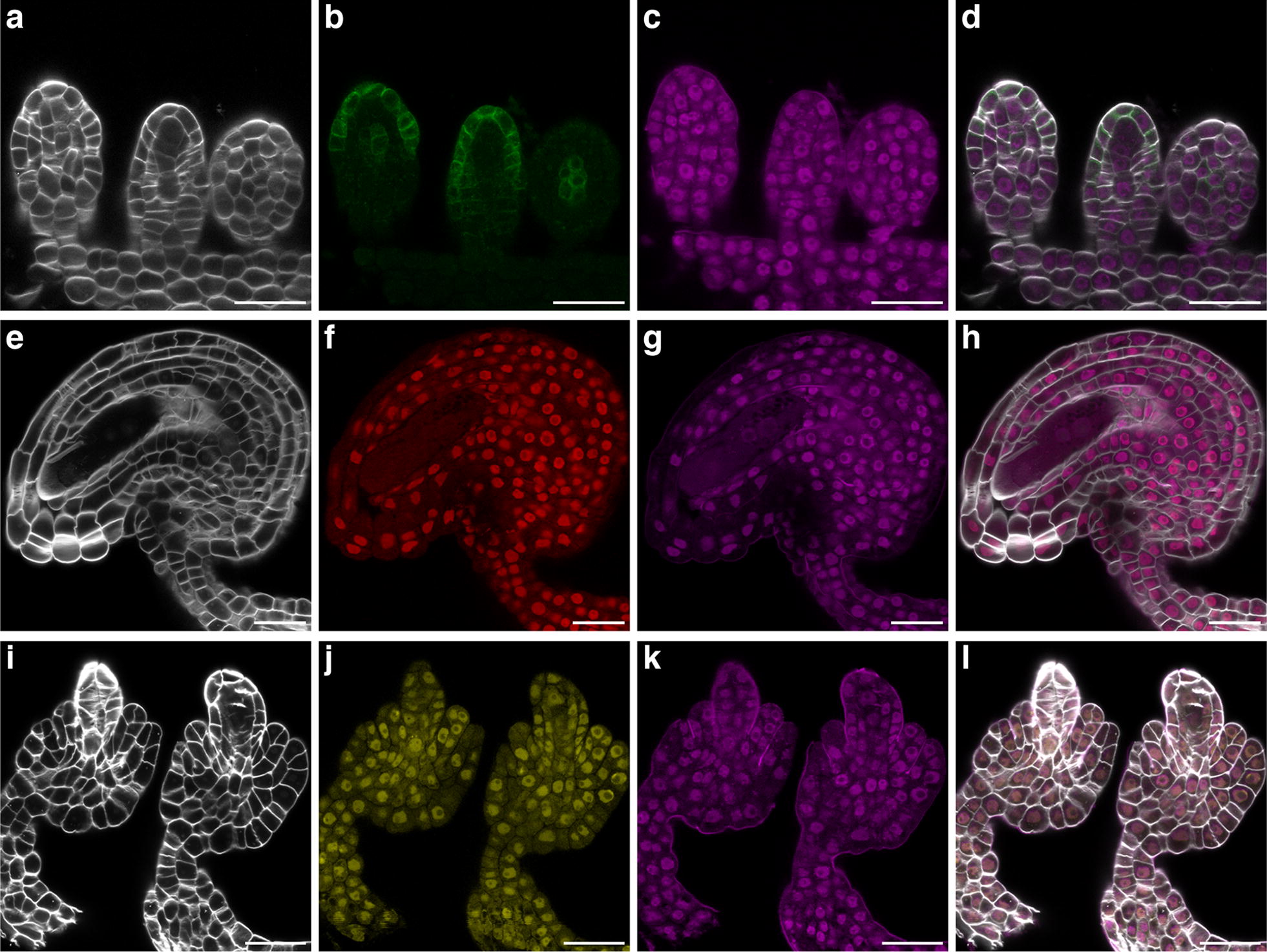
Compatibility with different fluorescent proteins. Mid-sagittal optical sections are shown. **a**–**d** Stage 2-II ovule from a Col-0 plant carrying the pPIN1::PIN1:GFP reporter [75]. **a** SR2200 stain. **b** Note the cell outlines marked by PIN1:GFP. **c** TO-PRO-3 iodide signal. **d** Merged channels. **e**–**h** Stage 3-V ovule from a Col-0 plant carrying a translational fusion of histone H2B to the fluorescent protein tdTomato under the control of the promoter of the ribosomal protein gene *P16* (At3g60245) (pP16::H2B:tdTomato) [76]. Similar arrangement of images as depicted in **a**–**d**. **f** Note the nuclear H2B:tdTomato signal. **i**–**l** Stage 2-V ovule from a Col-0 plant carrying a pP16::PCNA2:mCitrine reporter (PCNA2: At2g29570). Similar arrangement of images as depicted in **a**–**d**. **j** Note the nuclear PCNA2:mCitrine signal. Scale bars: 20 μm

Next, we addressed issues with mounting tissue on slides. In the original ClearSee protocol mounting of samples is directly performed in ClearSee [44]. We observed that the TO-PRO-3 stain was sensitive to ClearSee resulting in rapid disappearance of the staining during mounting and in a failure to image the complete 3D structure of ovules simultaneously co-stained with SR2200 and TO-PRO-3. To address the rapid fading of TO-PRO-3 signal we tested the Vectashield antifade reagent that is routinely used in fluorescence imaging [63]. We noticed that mounting in Vectashield ClearSee-treated ovules simultaneously stained with SR2200 and TO-PRO-3 preserved the nuclear and cell wall staining (Fig. 4c). Moreover, it also allowed imaging of multiple ovules from the same slide. Taken together, we obtained the best results by combining ClearSee, SR2200, and TO-PRO-3, followed by dissection and mounting the samples in Vectashield. Using the outlined method we routinely achieve deep imaging of mature ovules at cellular resolution with simultaneous labelling of cell outlines and nuclei. Moreover, we could confirm that this protocol is compatible with different FPs (Fig. 5).

**Fig. 6:**
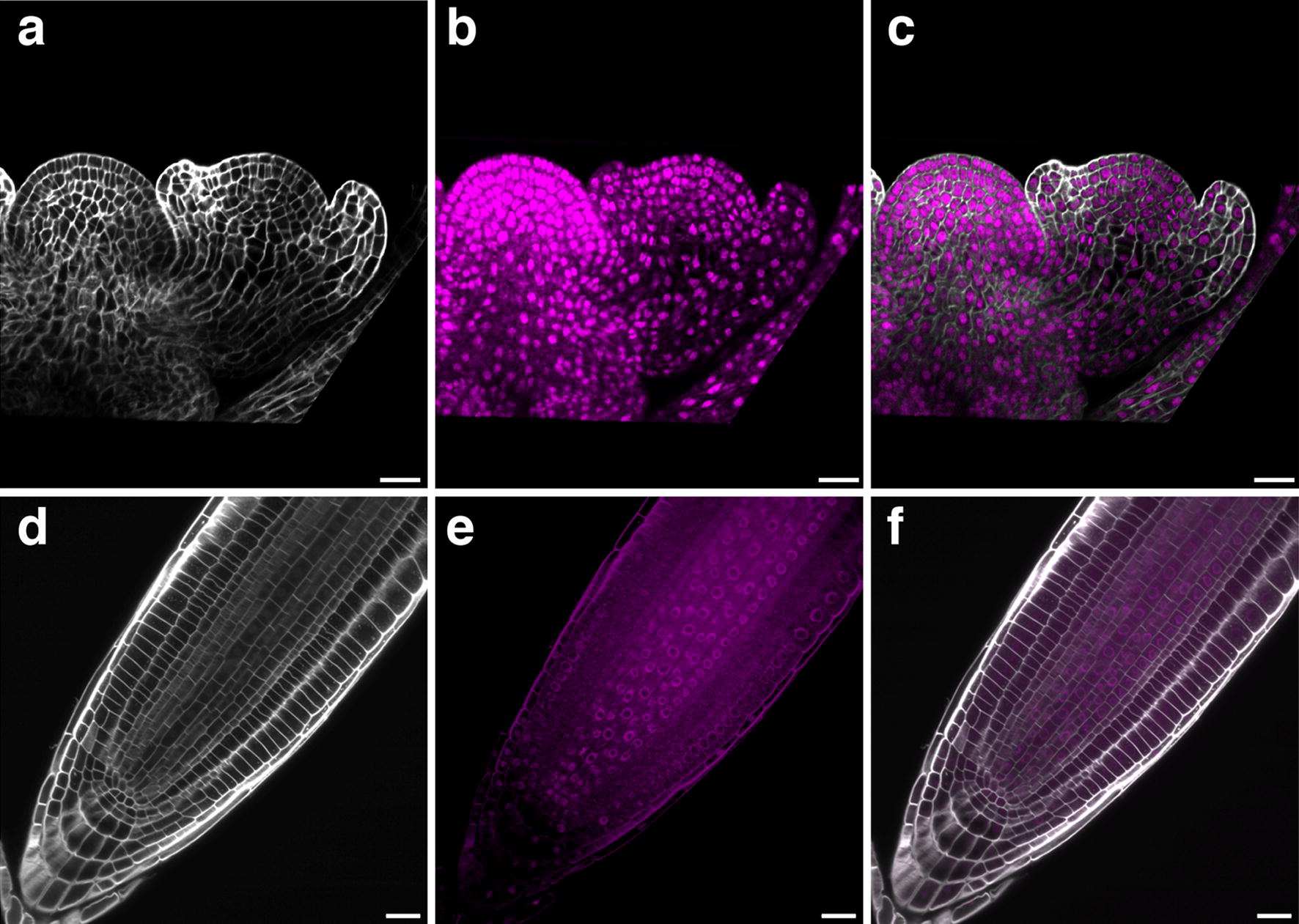
Applicability of the SR2000/TO-PRO-3 staining technique to different Arabidopsis tissues. Confocal micrographs depict mid-sagittal sections. **a**–**c** Inflorescence apex of 5-week-old plant with stage 3 floral meristem (stage according to [77]). **a** SR2200 staining. **b** TO-PRO-3 staining. **c** Merge. Both stains produce clear and specific signals. **d**–**f** Main root tip of a 7-day-old seedling. **d** SR2200 staining. **e** TO-PRO-3 staining. **f** Merge. Note TO-PRO-3 signal around the cells and diffuse signal within the cells. Scale bars: 20 μm

The Arabidopsis ovule represents a relatively small organ. To test if the technique can also be applied to bigger tissue we prepared inflorescence apices of 5-week old plants carrying young floral meristems and main root tips of 7-day seedlings. With respect to the inflorescence apex and floral meristem the technique worked surprisingly well and yielded images with cellular resolution (Fig. 6a–c). Staining the cell wall of root tip cells with SR2200 also worked well (Fig. 6d). However, staining root tip nuclei with TO-PRO-3 iodide resulted in poor stain penetration and diffuse intracellular signal.

**Fig. 7:**
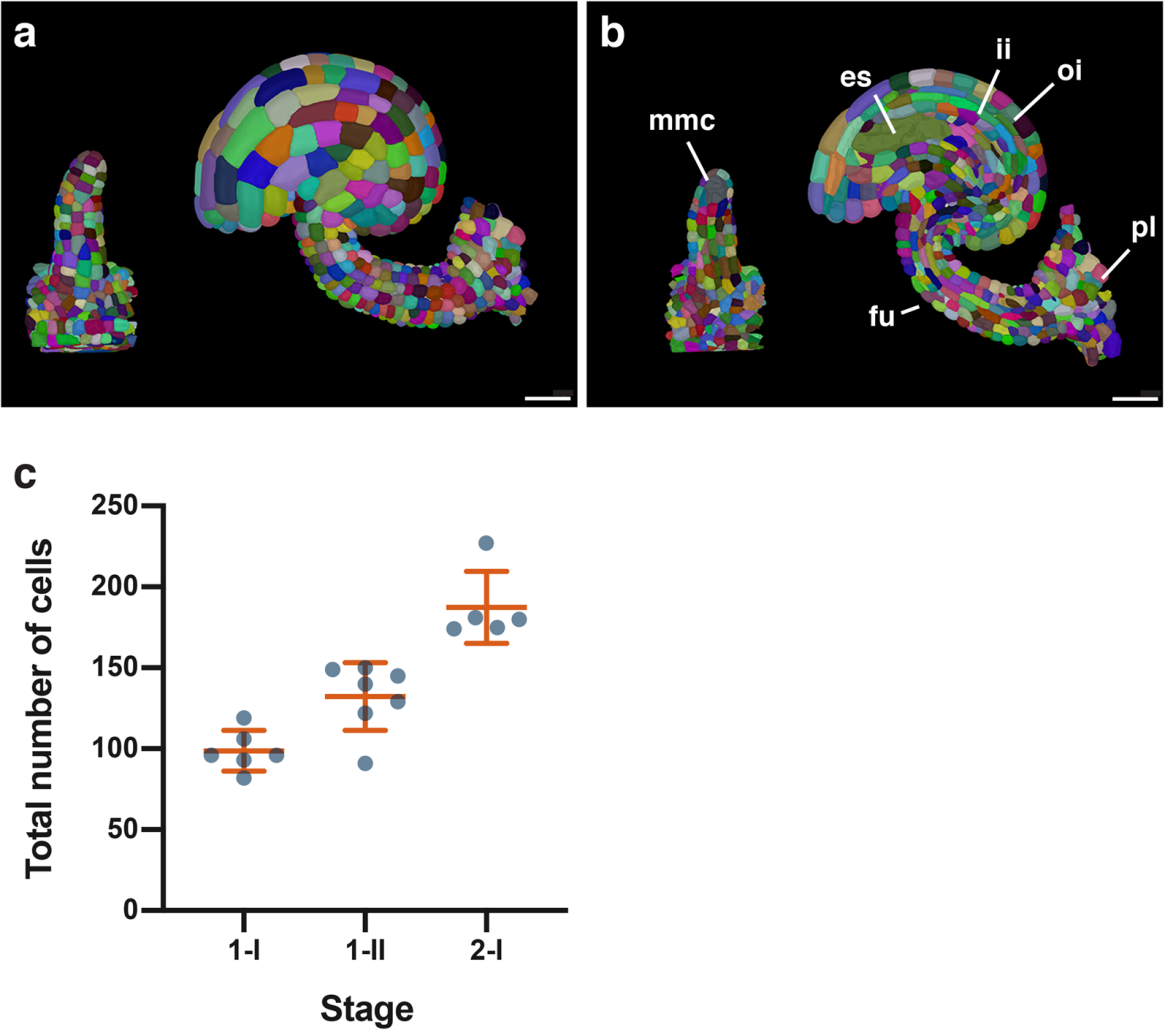
3D digital models with cellular resolution of wild-type Arabidopsis ovules. The models were obtained following deep-imaging of entire individual ovules. **a** Surface view. Left panel shows a stage 2-I ovule. Right panel depicts a stage 3 ovule. **b** Mid-sagittal sections through the 3D digital models depicted in **a**. **c** Number of cells per 3D digital ovule at three different stages during primordium formation. Mean ± SD is indicated. n = 5 for each stage. *ap* antipodal cells, *cc* central cell, *es* embryo sac, *fu* funiculus, *ii* inner integument, *mmc* megaspore mother cell, *oi* outer integument, *pl* placenta. Scale bars: 20 μm

We then assessed if z-stacks obtained by this imaging method can principally be used to generate completely segmented digital models of ovules. To this end we imported z-stacks of ovules of different stages into MorphographX [47] and processed the stacks using watershed-based segmentation followed by surface mesh formation. We found that the ITK watershed segmentation algorithm integrated in MorphographX was working well. However, hand correction by a domain expert was still required for optimal results. Nevertheless, we were able to rapidly generate digital 3D models of ovules with full cellular resolution (Fig. 7a, b). Two corresponding movies can be found in Additional files 1 and 2.

In a last step we tested the utility of such 3D models to gain quantitative cellular data. For example, it is not known how many cells contribute to a stage 1 ovule. Moreover, it is unclear whether cell proliferation in the ovule primordium follows a steady and continuous pattern or whether this process undergoes major stage-specific fluctuations. To provide preliminary first insight into these issues we imaged five wild-type (Col-0) ovules per stage for three stages covering primordium outgrowth and generated the respective 3D digital models. Next, we determined the total cell number of these models using MorphographX (Fig. 7c). We counted all the cells of the primordium that were found to reside above a plane that delineated the base of the ovule proper from the placenta. We found that at stage 1-I, the primordium features 98.67 ± 12.58 cells (mean ± SD) and at stage 1–II 132.3 ± 20.96 cells. At stage 2-I, when primordium length approaches its maximum [6], we observed 187.4 ± 22.35 cells. Within the limits of resolution of this method, the data further reveal that the increase in cell number across the measured stages is roughly linear indicating a continuous outgrowth of the primordium. The data demonstrate that the digital 3D ovule models obtained with the help of the SR2200/TO-PRO-3 staining method provide valuable tools for a quantitative analysis of ovule development.

## Discussion

Recent years have witnessed the development of several techniques aimed at clearing plant tissue and improving tissue penetration and cellular resolution of fluorescence microscopy [41–45, 64, 65]. Some protocols are more involved and may take weeks while others function best in certain tissues, such as leaves [45], or are not optimal for ovules, including the TOMEI technique that tends to yield aberrant ovule morphology [42, 43] (own observations).

Here we present a novel protocol that is rapid, simple, and allows reliable imaging of fixed Arabidopsis ovules of all developmental stages. The method combines two previously described techniques, ClearSee and SR2200 staining [17, 44], and includes several modifications and improvements for optimizing deep imaging of ovules with cellular resolution. As outlines of cells and nuclei are stained with different commercially available stains, the protocol simplifies downstream procedures. For example, it provides a high degree of freedom in choosing reporters of gene expression patterns or protein localization as 3D digital model generation does not require transgenic plants already expressing a FP-based marker that highlights cell shape. One disadvantage of the method relates to the observation that cells of the mature gametophyte cannot be stained well and need to be marked by other means. The detailed protocol with precise step-by-step instructions can be found in Additional file 3.

A key aspect of the modified protocol relates to combining the ClearSee protocol for tissue clearing with labelling the cell outline by the cell wall stain SR2200. For our purpose, SR2200 proved superior to other cell wall stains, including Direct Red 23 or Calcofluor, which have been reported to work well in combination with ClearSee in root tissue [46]. Another central point relates to nuclear staining with TO-PRO-3. TO-PRO-3 allows the ready detection of all nuclei including the nuclei of the developing gametophyte. Moreover, TO-PRO-3 is compatible with SR2200, the emission spectra of SR2200 and TO-PRO-3 do not overlap, and thus the two fluorescence signals can easily be collected in separate channels and analyzed independently. The unexpected rapid loss of signal intensity inTO-PRO-3 stained nuclei of ovules kept in ClearSee solution could be resolved by mounting the ovules in Vectashield upon clearing in ClearSee.

Deep imaging of ovules poses challenges with respect to gathering sufficient light and to spatial resolution. To address these issues we used a 63× glycerol objective with a numerical aperture of 1.30. In addition, we sought to create a maximally possible homogenous immersion system by minimizing differences between the refractive indices of the various media. In this context, Vectashield also proved a good compromise as we could stain nuclei with To-PRO-3 and still obtain acceptable spatial resolution. The average refractive index of the plant cell wall is around 1.42 [66]. Water has a refractive index of 1.33 and thus does not match the average refractive index of the cell wall. The ClearSee solution, however, has a refractive index of 1.41 [44]. Moreover, Vectashield [63] and the Leica Type G immersion liquid both have a refractive index of 1.45. Thus, the use of ClearSee and the Vectashield antifade reagent in combination with the 63× glycerol objective and the Leica Type G immersion liquid proved an acceptable and convenient solution to the problem.

Unexpectedly, the Vectashield mounting medium addressed some additional issues. Importantly, we could deep image different samples in a more consistent fashion. When ClearSee was used as mounting medium fewer ovules could be perfectly imaged in 3D. Moreover, Vectashield enabled us to image multiple ovules located on the same slide whereas ovules kept in ClearSee rapidly deteriorate, often already after having generated a single 3D stack of one particular ovule on the same slide. Even for Vectashield-mounted ovules optimal 3D stacks were obtained until up to about 2 h after mounting. However, good results could still be achieved until about 20 h after mounting. Finally, mounting in Vectashield reduced floating of samples.

The main focus of this research related to improving the imaging of ovules. However, we successfully applied this method to inflorescence apices and floral meristems. By contrast, imaging main root tips was only partially successful. While labelling cell contours with SR2200 worked well staining of this organ with TO-PRO-3 iodide resulted in only partial stain penetration and a more diffuse intracellular signal. This indicates that further improvements are necessary for imaging root tips or that TO-PRO-3 iodide is generally not suited for this tissue. Taken together, the results indicate wider applicability of the technique but its usefulness is currently restricted to certain tissues.

Digital 3D models with cellular resolution are crucial for the quantitative analysis of morphogenesis [23–25]. Our results suggest that this method is suitable for segmentation and the generation of digital 3D model of ovules at different developmental stages using MorphographX software. With the help of cellular 3D digital models we obtained some initial data on the cell proliferation patterns that generate the Arabidopsis ovule primordium. The modeling revealed that stage 2-I ovule primordia are made up of a relatively small group of slightly fewer than 200 cells. Moreover, the data indicate that ovule primordium formation appears to occur in a steadily progressive fashion. They provide proof-of-concept that interrogation of such 3D models can yield valuable quantitative insight into the cellular patterns underlying morphogenesis.

To obtain a fully segmented digital 3D model with complete cellular resolution still requires hand corrections performed by a domain expert. Thus, further work has to be done to improve the rapid, and ideally automated, generation of 3D digital ovule models, for example by improving image analysis of ovules and the segmentation algorithm, through deep learning approaches [67, 68].

## Conclusion

The availability of 3D digital models of organs with cellular resolution is essential for a quantitative analysis of tissue morphogenesis. We have established a new method for deep imaging of fixed and stained ovules of *Arabidopsis thaliana* from all developmental stages. By combining our improved imaging of ovules with presently available powerful 3D image analysis software tools, such as MorphographX, we established a straightforward pipeline that generates 3D digital models of ovules with cellular resolution. Such models will provide the foundation for a future quantitative analysis of ovule morphogenesis in *Arabidopsis thaliana*.

## Materials and methods

### Plant work

*Arabidopsis thaliana* (L.) Heynh. var. Columbia (Col-0) was used as wild-type strain. Plants were grown as described earlier [69].

### Clearing and staining of ovules

The mPS-PI staining was done as described [16]. Fixing and clearing of dissected ovules in ClearSee was done essentially as described [44]. DirectRed 23 and Calcofluor White staining in ClearSee was done as outlined in [46]. Staining with SR2200 was performed as described in [17] with minor modifications.

### Microscopy

Confocal laser scanning microscopy of tissue expressing GFP-based reporters was done using standard procedures, as described earlier [70, 71]. Confocal laser scanning microscopy of ovules stained with SR2200 and TO-PRO-3 iodide was performed on an upright Leica TCS SP8 X WLL2 HyVolution 2 (Leica Microsystems) equipped with GaAsP (HyD) detectors and a 63× glycerol objective (HC PL APO CS2 63×/1.30 GLYC, CORR CS2). Scan speed was at 400 Hz, line average between 2 and 4, and the digital zoom between 1 and 2. Laser power or gain was adjusted for z compensation to obtain an optimal z-stack. In case of the absence of GaAsP (HyD) detectors laser power and gain have to be adjusted according to the instrument at hand (Additional file 4). SR2200 fluorescence was excited with a 405 nm diode laser (50 mW) with a laser power ranging from 0.1 to 1.5% intensity and detected at 420 to 500 nm with the gain of the HyD detector set to 20. To-PRO-3 iodide fluorescence excitation was done at 642 nm with the white-light laser with a laser power ranging from 2 to 3.5% and detected at 655 to 720 nm with the gain of the HyD detector set to 200. Direct Red 23 fluorescence was excited using laser line 561 nm (white-light laser) with the laser power set to 5 to 8% and detected at 580 to 680 nm with the HyD gain set to 100. Calcofluor White fluorescence was excited using the 405 nm diode laser (50 mW) with the laser power set to 1 to 3% and detected at 420 to 490 nm with the HyD gain set to 100. For z-stacks 12-bit images were captured at a slice interval of 0.24 μm with optimized system resolution of 0.063 μm × 0.063 μm × 0.240 μm as final pixel size according to the Nyquist criterion. Scan speed was set to 400 Hz, the pinhole was set to 0.6 Airy units, line average was between 2 and 4, and the digital zoom was set between 1 and 2, as required. Laser power or gain was adjusted for z compensation to obtain an optimal z-stack.

Image acquisition parameters for pPIN1::PIN1:GFP, p16::H2B:tdTomato, and p16::PCNA2:mCitrine reporter lines: pPIN1::PIN1:GFP: SR2200; 405 diode laser 0.10%, HyD 420–480 nm, detector gain 10. GFP; 488 White laser 6%, HyD 500–590 nm, detector gain 100. TO-PRO-3; 642 nm White Laser 2%, HyD 660–720 nm, detector gain 100. p16::H2B:tdTomato: SR2200; 405 diode laser 0.10%, HyD 420–480 nm, detector gain 10. tdTomato; 554 White laser 4%, HyD 570–630 nm, detector gain 80. TO-PRO-3; 642 nm White Laser 2%, HyD 660–720 nm, detector gain 100. p16::PCNA2:mCitrine: SR2200; 405 diode laser 0.10%, HyD 420–480 nm, detector gain 10. mCitrine; 514 nm White Laser 5%, HyD 520–590 nm, detector gain 60. TO-PRO-3; 642 nm White Laser 2%, HyD 660–720 nm, detector gain 100. In each case sequential scanning was performed to avoid crosstalk between the spectra.

Images were adjusted for color and contrast using Adobe Photoshop CC (Adobe, San Jose, USA) or MorphographX [47] software (https://www.mpiz.mpg.de/MorphoGraphX). Scanning electron microscopy was performed essentially as reported previously [10, 72].

### Statistics

Statistical analysis was performed with PRISM8 software (GraphPad Software, San Diego, USA).

## Supplementary information

**Additional file 1.** Movie of a segmented mature ovule.

**Additional file 2.** Movie slicing through a segmented mature ovule.

**Additional file 3.** The detailed protocol.

**Additional file 4.** Comparison of GaAsP detectors versus PMTs.

## Data Availability

A PDF detailing the step-to-step procedure can be found in Additional file 3.
