# Supplementary material for: Protocol for rapid clearing and staining of fixed Arabidopsis ovules for improved imaging by confocal laser scanning microscopy
Source: Plant Methods. 2019 Oct 25;15:120. doi: 10.1186/s13007-019-0505-x (PMC6814113; doi:10.1186/s13007-019-0505-x)
Supplement: Supplementary file 3 — Additional file 3. The detailed protocol. [file 13007_2019_505_MOESM3_ESM.pdf]

Protocol for high-resolution 3D imaging and segmentation of ovules of *Arabidopsis thaliana*.

Rachele Tofanelli, Athul Vijayan, Kay Schneitz (2019)

Adapted from *Kurihara et al.* (2015), Musielak *et al*. (2015), and Ursache *et al.* (2018).

CHEMICALS

- PFA (Paraformaldehyde) (AppliChem, CAT/Order No: A3813,1000)
- Xylitol (Sigma-Aldrich, Cat No X3375, CAS-No: 87-99-0)
- Sodium Deoxycholate (Sigma-Aldrich, Cat No: 30970, CAS-No: 302-95-4)
- Urea (AppliChem, CAT No: A1049)
- SCRI Renaissance Stain 2200 (Renaissance Chemicals)
- TO-PRO™-3 Iodide (Thermo Fisher Scientific (Invitrogen), CAT No: T3605)
- VECTASHIELD® Antifade Mounting Medium (Vector Laboratories, CAT No: H-1000-10)

MATERIALS

- Coverslips 22x22 mm, 0.17 mm thickness (No. 1.5H, Paul Marienfeld GmbH & Ko. KG, Lauda-Königshofen, Germany, CAT No: 0107052)
- Insulin Syringes U-40 (1 ml/40 I.U.) (B. Braun Melsungen AG, Melsungen, Germany, CAT No: Inject® 40 Duo)
- Microscope Glass Slides 76x26x1 mm (Paul Marienfeld GmbH & Ko. KG, Lauda-Königshofen, Germany, CAT No: 1000000)
- Petri dish large 94/16 mm (Zefa, CAT No: 10029880)
- Petri dish small 35/10 mm (Opti-Lab GmbH, Munich, Germany, Petri dishes, CAT No: 6055567)
- Double sticky tape (Kaut-Bullinger, München, Germany, CAT No: 055446)

CLEARSEE SOLUTION

- Xylitol [final 10% (w/v)]
- Sodium Deoxycholate [final 15% (w/v)]
- Urea [final 25% (w/v)]
- Water to the final volume

NOTE: Mix the ClearSee solution for at least 30min to 1 hour on the magnetic stirrer until everything is completely dissolved!

FIXATIVE

1. Prepare 4% paraformaldehyde in 1x PBS solution to a final volume of 100 ml.
2. Heat and stir the solution to approximately 60°C. Take care that the solution does not boil! **Don't go over 70°C!**
3. The powder will not immediately dissolve. Slowly raise the pH by adding NaOH or KOH dropwise from a pipette until the solution clears
4. Once the paraformaldehyde is dissolved, recheck the pH, and adjust it with small amounts of HCl to approximately 6.9 pH
5. Cool down the solution before use

NOTE: The fixative can be aliquoted and kept at +4ºC or - 20Cº. Always use fresh PFA to prepare the fixative. The fixative can be stored for up to one week (4°C) or two weeks (-20°C)!

10x PBS Stock

Dissolve the following in 800ml distilled H2O.

1. 80g NaCl (1.37 M)
2. 2.0g KCl (27 mM)
3. 14.4g Na_2HPO_4 (100 mM)
4. 2.4g KH_2PO_4 (18 mM)
5. Adjust pH to 7.4
6. Adjust volume to 1L with additional distilled H2O
7. Sterilize by autoclaving
8. Prepare 1x PBS solution by diluting 10x PBS stock solution 1:10 in distilled H2O and adjust pH to 7.4.

STAINING SOLUTION: SR2200 and TO-PRO®-3

Prepare a combined staining solution containing 0.1% SR2200 and 1μM TO-PRO®-3 (1:1,000 dilution of 1 mM stock solution) in fresh 1x PBS.

NOTE: Avoid exposure to light. For best results, prepare fresh solution. SR2200 stock solution should be aliquoted to avoid repetitive handling of the original stock. The solution showed tendency to crystallization. Staining could be also done for individual stain rather than combined.

FIXATION AND CLEARING

1. Check the inflorescence and select the flowers at the proper stage.
2. Harvest the flower and place it under the stereomicroscope on a double-sided tape on the bottom of an inverted petri dish. Excise the carpel by using tweezers. With the help of a needle slightly open the carpels to expose the ovules.
3. Quickly transfer the pistils to a small petri dish with a double-sided tape fixed at the bottom and containing fixative. If necessary, pistils can be attached to the double-sided tape to prevent them from floating. The fixation is effective when the pistils sink to the bottom of the petri dish.
4. Fix for at least 1 to 2 hours at room temperature with gentle agitation or overnight at 4°C. Samples should be transferred to 1.5 ml microcentrifuge tubes containing fixative for overnight or long-term storage (up to one month).
5. If fixation was done at 4°C put the samples at room temperature for at least 30 min before proceeding
6. Carefully remove most of the fixative but make sure samples are still submerged. Don't let samples dry out!
7. Wash twice the fixed tissues for 1 min in 1 x PBS. **The washing step is very important to avoid the formation of precipitates that occur upon addition of ClearSee to the fixative!**
8. Transfer the fixed and washed carpels to a 1.5 ml microcentrifuge tube containing 1 ml ClearSee solution and clear them at room temperature overnight with gentle agitation. Overnight is usually sufficient. Slightly better results are obtained upon 2 to 3 days of clearing. Change the ClearSee solution after 2 days if samples are stored for extended periods of time. The carpels are stable in ClearSee for several weeks. After clearing proceed to the staining procedure.

STAINING PROCEDURE

1. Wash the cleared tissue for 1 minute in 1x PBS solution containing 0.1% SR2200 stain.
2. Transfer the pistils to another small petri dish filled with 1 ml of combined staining solution (0.1% SR2200 and TO-PRO®-3 (final dilution of 1:1,000)).
3. Stain at room temperature with gentle agitation for 20 minutes.
4. Wash the stained tissues for 1 minute in 1 x PBS.
5. Transfer the pistils into ClearSee solution for 20 minutes with gentle agitation for final clearing.
6. Proceed immediately with mounting.

NOTE: Use tweezers to transfer the pistil from one solution to another. Avoid mixing the solutions.

MOUNTING

1. Stick two coverslips with transparent nail polish on a microscopy slide leaving around 1.5 cm between them.
2. Place a drop of VECTASHIELD® antifade mounting medium on the slide.
3. Gently pick up a pistil from the ClearSee solution and place it on the slide with a minimum of ClearSee solution transferred.
4. Dissect the ovule from the pistil with the help of syringe needles.
5. Gently place a coverslip onto the sample and store the slide in a slide box at 4 °C.
6. Let the samples settle for 1 to 2 hours before imaging.

NOTE: For best results, imaging should be done 1 to 2 hours after mounting and on the same day. However, slides with samples mounted in VECTASHIELD® can be stored for up to a week with minor reduction in image quality.

GENERAL NOTES

- It is important to carry out every step under the stereomicroscope with minimal light exposure to avoid drying and shrinkage of ovules.
- Tissue in fixative can be stored for several weeks.
- Prior to staining samples can be stored in ClearSee solution for several days to optimize clearing.
- Do not store stained tissue in ClearSee solution for more than 2 hours prior to mounting in VECTASHIELD®) to avoid degradation of image quality.
- The protocol can be combined with fluorescent reporters. However, in such a case it is recommended to fix the samples for only 1 or 2 hours and perform clearing only overnight. Otherwise the fluorescent signal is weaker or lost completely.
- The support coverslips prevent the mounted ovules from squeezing. The coverslips thickness may not exceed the working distance of the objective used for imaging.

IMAGE ACQUISITION

Imaging is done with a confocal microscope. We use an upright Leica TCS SP8 X WLL2 HyVolution 2 (Leica Microsystem) equipped with HyD (GaAsP) detectors.

Cell wall

SR2200 excitation is done at 405 nm and its emission is detected at 420 - 500 nm with the HyD detector gain at 20. Laser power ranges from 0.1 to 1.5 % (50 mW diode laser).

Nuclei

TO-PRO™-3 Iodide excitation is done at 642 nm and its emission is detected at 655-720 nm with the HyD detector gain at 200. Laser power ranges from 2 to 3.5% (white-light laser).

Objective

63x glycerol objective (HC PL APO CS2 63x/1.30 GLYC)

Z-stacks

12-bit images are captured at a slice interval of 0.24 µm with optimized system resolution of 0.063 µm x 0.063 µm x 0.240 µm as final pixel size according to the Nyquist criterion. Scan speed is set to 400 Hz, the pinhole is set to 0.6 Airy units, line average should be between 2 and 4, and the digital zoom is set between 1 and 2. Laser power or gain may need to be adjusted for z compensation to obtain an optimal z-stack. Images can be adjusted for color and contrast using ImageJ/Fiji (https://fiji.sc), Adobe Photoshop (Adobe, San Jose, USA) or MorphographX (https://www.MorphoGraphX.org) software.

3D Segmentation in MorphographX

Segmentation is performed in MorphoGraphX (https://www.MorphoGraphX.org) software following the instructions documented in the user manual (https://www.mpipz.mpg.de/4085950/MGXUserManual.pdf).

The original stack is exported as a multilayered TIF file from ImageJ/Fiji (https://fiji.sc). Each channel is exported separately.

The TIF file of the 3D data set is loaded into MorphoGraphX by drag and drop into the main window. It will automatically load into “stack 1”. To improve the accuracy of 3D segmentation, raw data are processed to enhance the cell border signal and reduce the noise. To this end the data set is brightened `[Process/Stack/Filters/Brighten Darken]` by a value ranging from 2 to 3 and blurred `[Process/Stack/Filters/Gaussian Blur]` with a radius value ranging from 0.3 to 0.6 µm. Values can be adjusted according to the quality of the image.

3D segmentation is performed by auto-seeded ITK watershed libraries with the default threshold of 1500 `[Process/Stack/ITK/Segmentation/ITK Watershed Auto Seeded]`. The threshold value can be adjusted according to the results of the segmentation. Increase or decrease the threshold if the sample is over- or under-segmented, respectively. To identify problems with segmentation, compare with the original TIF file. In case of over-segmentation one original cell has been split into two or more segmented cells. Under-segmented cells are recognized by two or more original cells “fused” into one segmented cell.

After deleting the outside label (marking the “background” of the segmented cell) using `[Delete Picked Label in Volume]` from the volume tool bar, over-segmented cells are manually corrected with the color picker and bucket from the volume tool bar to fuse over-segmented cells. The

inside layers of the organ are checked using the clipping plane present in the view tab. The segmentation results are saved as TIF or mgxs files by selecting save from the file menu in stack 1. 3D meshes `[Process/Mesh/Creation/Marching Cubes 3D]` can be created from the segmented stack to extract volumetric data for further analysis.

REFERENCES

Kurihara, D., Mizuta, Y., Sato, Y., and Higashiyama, T. (2015). ClearSee: a rapid optical clearing reagent for whole-plant fluorescence imaging. Development 142, 4168-4179. doi: 10.1242/dev.127613.

Musielak, T. J., Schenkel, L., Kolb, M., Henschen, A., and Bayer, M. (2015). A simple and versatile cell wall staining protocol to study plant reproduction. Plant Reprod 28, 161-169. doi: 10.1007/s00497-015-0267-1.

Ursache, R., Andersen, T. G., Marhavý, P., and Geldner, N. (2018). A protocol for combining fluorescent proteins with histological stains for diverse cell wall components. Plant J 93, 399-412. doi: 10.1111/tpj.13784.
