# Supplementary material for: Protocol for rapid clearing and staining of fixed Arabidopsis ovules for improved imaging by confocal laser scanning microscopy
Source: Plant Methods. 2019 Oct 25;15:120. doi: 10.1186/s13007-019-0505-x (PMC6814113; doi:10.1186/s13007-019-0505-x)
Supplement: Supplementary file 4 — Additional file 4. Comparison of GaAsP detectors versus PMTs. [file 13007_2019_505_MOESM4_ESM.pdf]

Protocol for rapid clearing and staining of fixed Arabidopsis ovules for improved imaging by confocal laser scanning microscopy

Rachele Tofanelli, Athul Vijayan, Sebastian Scholz, and Kay Schneitz

Entwicklungsbiologie der Pflanzen, Wissenschaftszentrum Weihenstephan, Technische Universität München, Freising, Germany

Comparison between HyD and PMT detectors

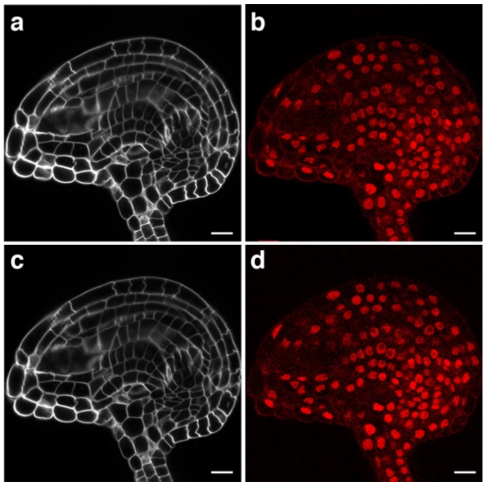

Confocal micrographs show mid-optical sections through fixed mature ovules from Col-0 plants carrying a pP16::H2BV:tdTomato reporter and stained with SR2200. Images were obtained using an upright Leica TCS SP8 X WLL2 HyVolution 2 (Leica Microsystems) equipped with GaAsP (HyD) detectors, PMTs, and a 63x glycerol objective (HC PL APO CS2 63x/1.30 GLYC, CORR CS2).

(**a,b**) Image acquisition with HyD detectors.

(**a**) SR2200: 405 diode laser 0.10%, HyD 420 nm – 480 nm, detector gain 10.

(**b**) tdTomato: 554 White laser 4%, HyD 570 nm – 630 nm, detector gain 80.

(**c,d**) Image acquisition with PMTs.

(**c**) SR2200: 405 diode laser 0.10%, PMT 420 nm – 480 nm, detector gain 600.

(**d**) tdTomato: 554 White laser 12%, PMT 570 nm – 630 nm, detector gain 800.
